# Development of Swallowing Function in Infants with Oral Feeding Difficulties

**DOI:** 10.1155/2020/5437376

**Published:** 2020-02-03

**Authors:** Changhun Han, Jaeho Shin, Ga Won Jeon

**Affiliations:** ^1^Department of Pediatrics, Inje University Busan Paik Hospital, Busan, Republic of Korea; ^2^Division of Pediatric Surgery, Department of Surgery, Inje University Busan Paik Hospital, Busan, Republic of Korea; ^3^Inje University College of Medicine, Busan, Republic of Korea

## Abstract

**Background:**

Discharge of preterm infants is often delayed because of their oral feeding difficulties. Independent oral feeding is the last obstacle to pass after managing acute and chronic morbidities. We conducted this study to determine the prevalence, characteristics, and risk factors of swallowing dysfunction and suggest proper interventions to reduce aspiration and chronic lung injury.

**Methods:**

Infants admitted to the neonatal intensive care unit (January 2016 to December 2018) who were performed modified barium swallow study due to oral feeding difficulties were enrolled. Modified barium swallow study was done ≥ postmenstrual age 37 weeks to limit radiation exposure. Clinical data were collected retrospectively. Swallowing dysfunction was defined as inadequate epiglottic closure, laryngeal penetration, or tracheal aspiration according to result of the modified barium swallow study.

**Results:**

Among a total of 54 infants enrolled, nine (16.7%) were term infants, 13 (24.1%) were late preterm infants (gestational age, 34-36 weeks), and 32 (59.3%) were early preterm infants (gestational age < 34 weeks). Gestational age and birth weight were smaller in infants with swallowing dysfunction. Total duration of mechanical ventilation and duration of invasive ventilation were longer in infants with swallowing dysfunction. The risk of swallowing dysfunction increased by 11.2 times for infants with gestational age < 29 weeks compared to infants with gestational age ≥ 29 weeks. Swallowing dysfunction was improved in most infants after they became matured. They showed different time and rate of maturation with the help of rehabilitation through swallow therapy and dietary modification with thickened formula.

**Conclusion:**

Preterm infants with gestational age < 29 weeks or with longer ventilation duration are at a higher risk of aspiration. Rehabilitation of swallow therapy and dietary modification with thickened formula can be helpful interventions to prevent aspiration and chronic lung injury and reassure parents until independent oral feeding is possible.

## 1. Introduction

Discharge of preterm infants from a neonatal intensive care unit (NICU) is often delayed because of their oral feeding difficulties. Independent oral feeding is the last obstacle to pass after managing acute and chronic morbidities associated with prematurity. Infant oral feeding is a relatively unexplored field. Inadequate oral feeding performance may not be taken as a serious problem. Particularly, term and late preterm infants usually are not admitted to a NICU or with short hospital stay. Thus, their oral feeding difficulties may be unrecognized or underestimated until hospital discharge [1]. During oral feeding, inspiration time is shortened and expiration time is prolonged, resulting in decreased minute ventilation and increased risk of aspiration. Furthermore, ineffective gas exchange of preterm infant may get even worse [2].

Independent oral feeding is achieved not only by swallowing itself but also by synchronization of sucking, swallowing, and breathing [3]. Their respiratory function will mature as preterm infants mature. Independent oral feeding is usually established near the time of discharge from NICU to home with coordination of sucking, swallowing, and breathing at a ratio of 1 : 1 : 1 or 2 : 2 : 1 [4].

However, some preterm infants may still have feeding desaturation near the time of discharge. It is difficult to figure out whether desaturation during oral feeding is caused by aspiration itself or other conditions such as sepsis, bronchopulmonary dysplasia (BPD), and necrotizing enterocolitis [5]. Thus, exact assessment of aspiration is crucial to provide proper interventions for the prevention of chronic aspiration and pulmonary dysfunction.

Modified barium swallow (MBS) study is a videofluoroscopic swallowing test to evaluate oropharyngeal swallowing function. MBS study is a standard diagnostic tool to evaluate oropharyngeal aspiration. It is more sensitive than upper gastrointestinal study (UGIS) [6]. MBS study is performed by rehabilitation pathologists. It is composed of oral phase, pharyngeal phase, and esophageal phase [7]. It can dynamically capture milk transfer from mouth to cervical esophagus. MBS study is preferred because of its relative short testing time, small amount of barium, and less radiation exposure compared to UGIS or esophagogram.

Thus, the objective of this study was to determine the prevalence, characteristics, and risk factors of swallowing dysfunction among infants who were referred for MBS study and suggest proper interventions to reduce aspiration and chronic lung injury.

## 2. Materials and Methods

### 2.1. Patients

The protocol of this study was reviewed and approved by the Institutional Review Board of Inje University Busan Paik Hospital (identification code: 19-0073) in accordance with the Declaration of Helsinki. The Institutional Review Board waived the need for informed consent for this retrospective chart review. Infants who were admitted to the NICU of Busan Paik Hospital and performed MBS study between January 2016 and December 2018 were enrolled. Oral feeding was initiated when the infant had oromotor cues around postmenstrual age 34 weeks. MBS study was done in infants with oral feeding difficulties no younger than postmenstrual age 37 weeks. Oral feeding difficulties included SpO_2_ < 80% for 15 seconds with bradycardia during oral feeding by the nurse more than four out of eight feedings per day. Infants who had anatomical anomalies in organs involved in suck and swallow mechanism such as cleft lip, cleft palate, or tracheoesophageal fistula were excluded as these conditions could be confounding factors by interfering with independent oral feeding, making them unsuitable for evaluating the development of swallowing function.

Enrolled infants were allocated to two groups according to the result of MBS study: infants with swallowing dysfunction and infants without swallowing dysfunction. They were stratified into three groups according to gestational age: early preterm infants, gestational age < 34 weeks; late preterm infants, gestational age of 34-36 weeks; and term infants, gestational age of 37-41 weeks.

### 2.2. Study Protocol

Clinical data were collected retrospectively from medical records. A fluoroscope (Artis Zee Ceiling System, Siemens Healthcare, Munich, Germany) was used with duration of less than two minutes. It was operated during the initial swallowing to reduce radiation exposure according to Weir et al. [8]. MBS study was done with milk bottle filled with 10 mL of barium added to 30 mL of formula at a ratio of 1 : 3. Infant was placed in a semiupright sitting position at 45 degrees.

In this study, according to the result of MBS study, inadequate epiglottic closure, laryngeal penetration, and tracheal aspiration were classified as swallowing dysfunction that could result in respiratory aspiration [9].

The definition of inadequate epiglottic closure was delayed in epiglottic closure when swallowing was initiated. Laryngeal penetration was detected by the presence of barium underneath the epiglottis, but not in the true vocal cords. Tracheal aspiration was detected by the presence of barium below the level of the true vocal cords [10].

To identify the factors that might influence swallowing dysfunction, various perinatal and postnatal variables were evaluated. Demographic factors included gestational age, birth weight, gender, Apgar score, small for gestational age (SGA), clinical risk index for babies (CRIB) II score, antenatal steroid therapy, gestational diabetes mellitus, maternal pregnancy-induced hypertension, and histologically confirmed chorioamnionitis. Outcomes associated with prematurity included respiratory distress syndrome, duration of mechanical ventilation, BPD, severity of BPD, patent ductus arteriosus, high-grade intraventricular hemorrhage (≥grade 3), periventricular leukomalacia, high-stage retinopathy of prematurity (≥stage 2), necrotizing enterocolitis (≥stage 2), and duration of hospital stay.

SGA was defined when birth weight was less than the tenth percentile according to the new fetal-infant growth chart [11]. Respiratory distress syndrome was diagnosed by the attending physician based on the infant's respiratory symptom, chest radiography, and blood gas analysis [12]. BPD was defined as an oxygen dependency at postmenstrual age 36 weeks with oxygen treatment for at least the first 28 days of life [13]. This disorder was categorized to three groups by severity: mild, moderate, or severe BPD.

We continued current oral feeding with standard formula if the infant only had laryngeal penetration with mild symptom. The infant was fed leaning forwardly to prevent tracheal aspiration and discharged with home monitoring by pulse oximetry. Infants with tracheal aspiration were discharged with dietary management using cornstarch-thickened formula (Novalac AR®, UP International S.A, France). Some severe infants with tracheal aspiration were discharged with tube feeding. All infants who were discharged with dietary management or tube feeding underwent rehabilitation of swallow therapy.

### 2.3. Statistical Analysis

The chi-square test or Fisher's exact test was performed for dichotomous outcome data while *t*-test or Mann-Whitney *U* test was used for continuous data. Odds ratio with 95% confidence intervals using simple logistic regression analysis and coefficients (*β*) using multiple regression analysis were used to evaluate factors that might influence swallowing dysfunction. All statistical analyses were performed using IBM SPSS version 25.0 (IBM Corp., Armonk, NY, USA). *p* values < 0.05 were considered significant. Data are given as mean ± standard deviation.

## 3. Results

### 3.1. Swallowing Dysfunction according to Gestational Age

Among a total of 54 infants enrolled, nine (16.7%) were term infants, 13 (24.1%) were late preterm infants, and 32 (59.3%) were early preterm infants (Table 1). Among infants referred for MBS study, 44.4% (4/9) of term infants, 30.8% (4/13) of late preterm infants, and 59.4% (19/32) of early preterm infants had swallowing dysfunction.

Among 27 infants with swallowing dysfunction, four term infants had cerebral palsy. Two of them improved after dietary management with thickened formula. The remaining two infants continued tube feeding. Among four late preterm infants with swallowing dysfunction, one infant had mild developmental delay. The remaining three infants showed normal development. Their symptoms were improved markedly after dietary management with thickened formula. Among 19 early preterm infants with swallowing dysfunction, three of them who had only laryngeal penetration with mild feeding desaturation continued their oral feeding leaning forwardly. They were discharged with home monitoring using pulse oximetry. We changed the formula to thickened formula for 11 infants along with swallow therapy. Their symptoms were improved at the time of discharge from a NICU. Five of them with severe swallowing difficulty were discharged with tube feeding along with rehabilitation of swallow therapy.

We did not routinely perform follow-up MBS study if infants' symptoms associated with feeding were improved to limit subsequent radiation exposure. We performed follow-up MBS study for five early preterm infants discharged with tube feeding in a month after rehabilitation of swallow therapy. Three of them did not show aspiration anymore. One infant still showed aspiration in follow-up MBS study. However, she did not have choking episode when fed with thickened formula. Thus, bottle feeding was successful for these four infants. One of five tube-fed infants showed aspiration on subsequent study. He continued tube feeding and was confirmed to have cerebral palsy.

### 3.2. Characteristics of Preterm Infants with Swallowing Dysfunction

Gestational age and birth weight were smaller in the swallowing dysfunction group (30^+4^ ± 3^+2^ weeks vs. 33 ± 2^+5^ weeks, *p* = 0.010; 1595 ± 864 g vs. 2109 ± 900 g, *p* = 0.047, respectively) (Table 2). CRIB II was higher in the swallowing dysfunction group (5.4 ± 4.3 vs. 2.5 ± 1.8, *p* = 0.004). Sex ratio, SGA, or Apgar score (at one or five minutes) was not significantly different between the two groups. Administration of antenatal corticosteroids, maternal gestational diabetes mellitus, pregnancy-induced hypertension, or chorioamnionitis was not significantly different between the two groups either.

The incidence of respiratory distress syndrome was similar between the two groups. However, surfactant redosing was more frequent in the swallowing dysfunction group (8 (34.8%) vs. 2 (9.1%), *p* = 0.038). Total duration of mechanical ventilation (17 ± 24 days vs. 5 ± 9 days, *p* = 0.025) and duration of invasive ventilation (14 ± 20 days vs. 3 ± 5days, *p* = 0.015) were longer in the swallowing dysfunction group. Incidence of BPD, patent ductus arteriosus, intraventricular hemorrhage (≥grade 3), periventricular leukomalacia, retinopathy of prematurity (≥stage 2), or necrotizing enterocolitis (≥stage 2) and duration of hospital stay were similar between the two groups. Postmenstrual age at the time of MBS was also similar between the two groups (40 ± 2^+4^ weeks vs. 39^+4^ ± 2^+2^ weeks, *p* = 0.586).

### 3.3. Risk Factors of Swallowing Dysfunction in Preterm Infants

The risk of swallowing dysfunction increased by 11.2 times (95% confidence intervals: 1.264-99.267, *p* = 0.030) for infants with gestational age < 29 weeks compared to infants with gestational age ≥ 29 weeks (Table 3). Multiple regression analysis revealed that swallowing dysfunction decreased by 0.771 times with an increase in gestational age of one week (coefficients (*β*) = −0.260, standard error = 0.111, *p* = 0.019), suggesting that oral feeding skills would be more mature with increasing gestational age at birth.

## 4. Discussion

In this study, about 50% of preterm infants and about 60% of early preterm infants who were performed MBS study due to feeding desaturation had swallowing dysfunction. The prevalence of neonatal swallowing dysfunction is unknown yet because swallowing dysfunction is based on symptom and numerous phases are needed for independent oral feeding. No single symptom can imply swallowing dysfunction. These symptoms can be subjective. Thus, caregivers are prone to considering these symptoms as nonswallowing problems. Independent oral feeding needs numerous phases, including sucking, swallowing, maturation of sucking-and-swallowing, maturation of respiratory system, and coordination of sucking-swallowing-and-breathing. Lee et al. [14] have reported that 70% of very low birth weight infants who are referred for MBS study have feeding problems and 30% of very low birth weight infants have swallowing dysfunction. Jadcherla et al. [3] have shown that 24.5% of very low birth weight infants and up to 10.5% of preterm infants have feeding problems. Mercado-Deane et al. [15] have revealed that up to 26% of preterm infants have dysphagia.

Oral feeding in preterm infants with BPD is challenging because preterm infants already have respiratory problems such as decreased minute ventilation. Minute ventilation is even decreased during oral feeding [16, 17]. Apnea or desaturation events during feeding is more frequent in infants with BPD compared to that in those without BPD. In addition, infants with BPD spend more time for oral feeding [18, 19]. Up to 30% of preterm infants with persistent feeding dysfunction at the age of less than one year have BPD [15]. In our study, the incidence of BPD was higher in infants with swallowing dysfunction, although the difference was not statistically significant. However, the duration of mechanical ventilation, especially the duration of invasive ventilation was much longer in infants with swallowing dysfunction. We think that although BPD could be subdivided to mild, moderate, and severe forms, such subdivision cannot reflect diverse lung conditions. Hence, ventilation duration or invasive ventilation duration can be a more sensitive variable than BPD reflecting lung conditions.

In our study, two term infants and one early preterm infant with prolonged swallowing dysfunction were confirmed to have cerebral palsy. Frequent aspiration and dysphagia in neonatal period can be a predictor of neurodevelopmental delay, and infants with developmental delay usually have prolonged aspiration or feeding problems in neonatal period, vice versa [20]. So, we need to check infants with prolonged feeding problems whether there are neurologic complications.

Preterm infants' nutritive sucking pattern usually develops to a pattern similar to term infants, which is the fifth stage of nutritive sucking pattern. It is the most mature stage with rhythmic, well-defined suction and expression [2]. Term infants' sucking pattern has to be fully developed. Thus, caregivers need to pay attention to swallowing dysfunction occurring in term infants. Feeding desaturation in term infants is sometimes associated with brain, heart, or lung problem [21, 22]. Among term infants with swallowing dysfunction in the present study, one had pulmonary sequestration and one had skeletal dysplasia with small thorax and small nasal cavity. Thus, problems in the respiratory system might cause swallowing dysfunction, although infants may not have any anomalies in organs involved in sucking and swallowing mechanism directly. It means that maturation of the respiratory system is also important for independent oral feeding.

Davis et al. [23] have reported that among preterm infants who were performed MBS study, 28% (42/148) are late preterm infants and 57% of these infants have swallowing dysfunction. In the present study, among preterm infants who were performed MBS study, 28.9% (13/45) of them were late preterm infants and 30.8% of them had swallowing dysfunction. This emphasizes that oral feeding immaturity is sometimes underestimated in late preterm infants, although these infants are at a high risk of aspiration. Swallowing dysfunction of most of late preterm infants was improved after dietary modification with thickened formula. They also showed normal development. Thus, their swallowing dysfunction is a matter of oral feeding skill development.

Gestational age and birth weight were smaller in infants with swallowing dysfunction in the present study. Gestational age at birth was an important risk factor to predict swallowing dysfunction. Preterm infants with gestational age < 29 weeks had 11 times higher risk of swallowing dysfunction in this study. Therefore, we need to thoroughly check preterm infants with gestational age < 29 weeks to determine whether there are feeding problems.

Postmenstrual age at the time of MBS was not significantly different between the two groups in this study. We do not routinely perform MBS study. We usually wait until postmenstrual age 37 weeks to limit radiation exposure by MBS study. Infants with swallowing dysfunction had younger gestational age at birth. However, postmenstrual age at MBS was similar, meaning that the development of oral feeding skills is influenced by the gestational age at birth more than by postmenstrual age [14, 24]. However, according to other studies, the development of oral feeding skills depends on postmenstrual age rather than gestational age at birth [25, 26]. In our study, swallowing dysfunction was improved as infants became more mature, although they showed different time and rate of maturation.

The proper time to initiate oral feeding is known to be postmenstrual age 34 weeks. However, the possible earliest time to initiate oral feeding is currently unknown. The time and the rate of maturation of oral feeding skills are different [27]. Though the feeding performance of preterm infants reached only the tenth percentile of the healthy term infants at postmenstrual age 34-35 weeks, it matured at postmenstrual age 38-40 weeks by the noninvasive evaluation of swallowing sound [28]. Early initiation of oral feeding with sufficient time for training can enhance oral feeding skills and lead to earlier independent oral feeding than late initiation of oral feeding [29]. It has been suggested that oral feeding can be initiated at the stage 1 or 2 of the “five stages of sucking” [29].

Rehabilitation of swallow therapy can advance musculature related to sucking, promote mature sucking stages, and generate higher sucking amplitude [30]. It can also help neural maturation of sucking, swallowing, and respiration. Finally, it can help the coordination and synchronization of swallowing processes. In our study, infants underwent swallow therapy that improved their oral feeding skills. In mild cases, we continued bottle feeding and educated parents about feeding position with leaning forwardly to prevent tracheal aspiration. Sometimes thickened formula were used. Some were discharged with home monitoring. This protocol was good for parental reassurance and better compliance.

Thickened formula is based on adding thickening agents to increase viscosity and to decrease regurgitation [31]. Cornstarch-thickened formula is widely used in infants with gastroesophageal reflux [32]. Thickened formula was effective for improving swallowing dysfunction and parental reassurance in the present study. As thickened formula is not commonly used for infants with swallowing dysfunction, the mechanism involved in its reduction of feeding desaturation is currently unknown. Further study is needed.

There are several limitations in this study. Though the postmenstrual age at the time of MBS study was not different, the wide range of postmenstrual age of MBS study is one of the limitations of the retrospective study. Incidence of BPD, intraventricular hemorrhage, or retinopathy of prematurity was not different between the two groups. This might be due to the relative small sample size.

## 5. Conclusions

In conclusion, about 60% of early preterm infants referred for MBS study because of feeding desaturation had swallowing dysfunction. Late preterm infants with normal development also had swallowing dysfunction. Preterm infants with gestational age < 29 weeks or with longer ventilation duration are at a higher risk of aspiration. Thus, we need to check them in detail to determine whether there are feeding problems. Most of swallowing dysfunctions are improved as infant matures. However, chronic lung damage due to frequent aspiration should be prevented until independent oral feeding is possible. Rehabilitation of swallow therapy and dietary modification with thickened formula can be helpful interventions to prevent aspiration and chronic lung injury and reassure parents.

## Figures and Tables

**Table 1:** Swallowing dysfunction according to gestational age.

MBS study	Total (*n* = 54)	Term (*n* = 9)	Late preterm (*n* = 13)	Early preterm (*n* = 32)
Swallowing dysfunction	27	4	4	19

Management	Continue oral feeding with standard formula			3 (with laryngeal penetration)
	Change to cornstarch-thickened formula	2	3	11
	Tube feeding	2 (CP)	1 (with mild developmental delay)	5 (one with CP)

MBS: modified barium swallow; CP: cerebral palsy.

**Table 2:** Characteristics of preterm infants with swallowing dysfunction.

	Infants with swallowing dysfunction (*n* = 23)	Infants without swallowing dysfunction (*n* = 22)	*p* value
Gestational age (week^+day^)	30^+4^ ± 3^+2^	33 ± 2^+5^	0.010
Birth weight (g)	1595 ± 864	2109 ± 900	0.047
Male, *n* (%)	13 (56.5)	15 (68.2)	0.420
SGA, *n* (%)	5 (21.7)	2 (9.1)	0.242
Apgar score at 1 min	5.6 ± 1.9	5.7 ± 1.6	0.892
Apgar score at 5 min	7.3 ± 1.5	7.9 ± 1.0	0.116
CRIB II	5.4 ± 4.3	2.5 ± 1.8	0.004
Antenatal corticosteroids, *n* (%)	17 (73.9)	14 (63.6)	0.457
Maternal GDM, *n* (%)	2 (8.7)	5 (22.7)	0.194
Maternal PIH, *n* (%)	4 (17.4)	5 (22.7)	0.655
Chorioamnionitis, *n* (%)	5 (21.7)	5 (22.7)	0.936
RDS, *n* (%)	20 (87.0)	15 (68.2)	0.130
Surfactant redosing, *n* (%)	8 (34.8)	2 (9.1)	0.038
Duration of mechanical ventilation (days)	17 ± 24	5 ± 9	0.025
Duration of invasive ventilation (days)	14 ± 20	3 ± 5	0.015
BPD, *n* (%)	7 (30.4)	3 (13.6)	0.175
BPD (moderate to severe), *n* (%)	5 (21.7)	2 (9.1)	0.242
Ligation of PDA, *n* (%)	2 (8.7)	2 (9.1)	0.963
IVH (≥grade 3), *n* (%)	3 (13.0)	0 (0.0)	0.080
PVL, *n* (%)	2 (8.7)	2 (9.1)	0.963
ROP (≥stage 2), *n* (%)	3 (13.0)	0 (0.0)	0.080
NEC (≥stage 2), *n* (%)	0 (0.0)	0 (0.0)	>0.99
Hospital stay (days)	78 ± 39	61 ± 31	0.108
PMA at the time of MBS (week^+day^)	40 ± 2^+4^	39^+4^ ± 2^+2^	0.586

SGA: small for gestational age; CRIB: clinical risk index for babies; GDM: gestational diabetes mellitus; PIH: pregnancy-induced hypertension; RDS: respiratory distress syndrome; BPD: bronchopulmonary dysplasia; PDA: patent ductus arteriosus; IVH: intraventricular hemorrhage; PVL: periventricular leukomalacia; ROP: retinopathy of prematurity; NEC: necrotizing enterocolitis; PMA: postmenstrual age; MBS: modified barium swallow.

**Table 3:** Risk factors of swallowing dysfunction in preterm infant (by logistic regression analysis).

	Coefficient (*β*)	Standard error	*p* value	OR	95% CI
Gestational age (<29 vs. ≥29 weeks)	2.416	1.113	0.030	11.200	1.264-99.267

OR: odds ratio; CI: confidence interval.

## Data Availability

The data used to support the findings of this study are included within the article.

## Supplementary Materials

Supplementary Materials Patient flowchart. Among a total of 54 infants referred for MBS study, 27 infants had swallowing dysfunction. Four term infants with swallowing dysfunction had cerebral palsy. Two of them improved after dietary management with thickened formula. The remaining two infants continued tube feeding. Among four late preterm infants with swallowing dysfunction, three infants with normal development improved after dietary management with thickened formula. The remaining one infant with mild developmental delay continued tube feeding, who improved in follow-up MBS study. Among 19 early preterm infants with swallowing dysfunction, three infants who had only laryngeal penetration with mild feeding desaturation continued their oral feeding. Eleven infants improved after dietary management with thickened formula. The remaining five infants with severe swallowing dysfunction were discharged with tube feeding. Four of them improved in follow-up MBS study. One of them with cerebral palsy continued tube feeding.

## References

[1] Lau C. (2015). Development of suck and swallow mechanisms in infants. *Annals of Nutrition & Metabolism*.

[2] Lau C. (2016). Development of infant oral feeding skills: what do we know?. *The American Journal of Clinical Nutrition*.

[3] Jadcherla S. (2016). Dysphagia in the high-risk infant: potential factors and mechanisms. *The American Journal of Clinical Nutrition*.

[4] Fucile S., Phillips S., Bishop K., Jackson M., Yuzdepski T., Dow K. (2019). Identification of a pivotal period in the oral feeding progression of preterm infants. *American Journal of Perinatology*.

[5] Gianni M. L., Sannino P., Bezze E. (2017). Usefulness of the infant driven scale in the early identification of preterm infants at risk for delayed oral feeding independency. *Early Human Development*.

[6] Vazquez J. L., Buonomo C. (1999). Feeding difficulties in the first days of life: findings on upper gastrointestinal series and the role of the videofluoroscopic swallowing study. *Pediatric Radiology*.

[7] Watts S., Gaziano J., Jacobs J., Richter J. (2019). Improving the diagnostic capability of the modified barium swallow study through standardization of an esophageal sweep protocol. *Dysphagia*.

[8] Weir K. A., McMahon S. M., Long G. (2007). Radiation doses to children during modified barium swallow studies. *Pediatric Radiology*.

[9] Kahrilas P. J., Lin S., Rademaker A. W., Logemann J. A. (1997). Impaired deglutitive airway protection: a videofluoroscopic analysis of severity and mechanism. *Gastroenterology*.

[10] Brady S., Donzelli J. (2013). The modified barium swallow and the functional endoscopic evaluation of swallowing. *Otolaryngologic Clinics of North America*.

[11] Fenton T. R. (2003). A new growth chart for preterm babies: Babson and Benda's chart updated with recent data and a new format. *BMC Pediatrics*.

[12] Jobe A. (1983). Respiratory distress syndrome--new therapeutic approaches to a complex pathophysiology. *Advances in Pediatrics*.

[13] Kinsella J. P., Greenough A., Abman S. H. (2006). Bronchopulmonary dysplasia. *The Lancet*.

[14] Lee J. H., Chang Y. S., Yoo H. S. (2011). Swallowing dysfunction in very low birth weight infants with oral feeding desaturation. *World Journal of Pediatrics*.

[15] Mercado-Deane M. G., Burton E. M., Harlow S. A. (2001). Swallowing dysfunction in infants less than 1 year of age. *Pediatric Radiology*.

[16] Gewolb I. H., Bosma J. F., Taciak V. L., Vice F. L. (2001). Abnormal developmental patterns of suck and swallow rhythms during feeding in preterm infants with bronchopulmonary dysplasia. *Developmental Medicine and Child Neurology*.

[17] Reynolds E. W., Grider D., Caldwell R., Capilouto G., Patwardhan A., Charnigo R. (2018). Effects of Bronchopulmonary Dysplasia on Swallow:Breath Interaction and Phase of Respiration with Swallow During Non-nutritive Suck. *Journal of Nature and Science*.

[18] Wang L. Y., Luo H. J., Hsieh W. S. (2010). Severity of bronchopulmonary dysplasia and increased risk of feeding desaturation and growth delay in very low birth weight preterm infants. *Pediatric Pulmonology*.

[19] Garg M., Kurzner S. I., Bautista D. B., Keens T. G. (1988). Clinically unsuspected hypoxia during sleep and feeding in infants with bronchopulmonary dysplasia. *Pediatrics*.

[20] Raol N., Schrepfer T., Hartnick C. (2018). Aspiration and dysphagia in the neonatal patient. *Clinics in Perinatology*.

[21] Karsch E., Irving S. Y., Aylward B. S., Mahle W. T. (2017). The prevalence and effects of aspiration among neonates at the time of discharge. *Cardiology in the Young*.

[22] Hawdon J. M., Beauregard N., Slattery J., Kennedy G. (2000). Identification of neonates at risk of developing feeding problems in infancy. *Developmental Medicine and Child Neurology*.

[23] Davis N. L., Liu A., Rhein L. (2013). Feeding immaturity in preterm neonates: risk factors for oropharyngeal aspiration and timing of maturation. *Journal of Pediatric Gastroenterology and Nutrition*.

[24] Amaizu N., Shulman R., Schanler R., Lau C. (2008). Maturation of oral feeding skills in preterm infants. *Acta Paediatrica*.

[25] Qureshi M. A., Vice F. L., Taciak V. L., Bosma J. F., Gewolb I. H. (2002). Changes in rhythmic suckle feeding patterns in term infants in the first month of life. *Developmental Medicine and Child Neurology*.

[26] Vice F. L., Gewolb I. H. (2008). Respiratory patterns and strategies during feeding in preterm infants. *Developmental Medicine and Child Neurology*.

[27] John H. B., Suraj C., Padankatti S. M., Sebastian T., Rajapandian E. (2019). Nonnutritive sucking at the mother's breast facilitates oral feeding skills in premature infants: a pilot study. *Advances in Neonatal Care*.

[28] Ince D. A., Ecevit A., Acar B. O. (2014). Noninvasive evaluation of swallowing sound is an effective way of diagnosing feeding maturation in newborn infants. *Acta Paediatrica*.

[29] Lau C., Alagugurusamy R., Schanler R. J., Smith E. O., Shulman R. J. (2000). Characterization of the developmental stages of sucking in preterm infants during bottle feeding. *Acta Paediatrica*.

[30] Fucile S., McFarland D. H., Gisel E. G., Lau C. (2012). Oral and nonoral sensorimotor interventions facilitate suck-swallow-respiration functions and their coordination in preterm infants. *Early Human Development*.

[31] Salvatore S., Savino F., Singendonk M. (2018). Thickened infant formula: what to know. *Nutrition*.

[32] Chao H. C., Vandenplas Y. (2007). Comparison of the effect of a cornstarch thickened formula and strengthened regular formula on regurgitation, gastric emptying and weight gain in infantile regurgitation. *Diseases of the Esophagus*.

