# Supplementary figures and images for: Development of Swallowing Function in Infants with Oral Feeding Difficulties
Source: Int J Pediatr. 2020 Feb 3;2020:5437376. doi: 10.1155/2020/5437376 (PMC7023804; doi:10.1155/2020/5437376)

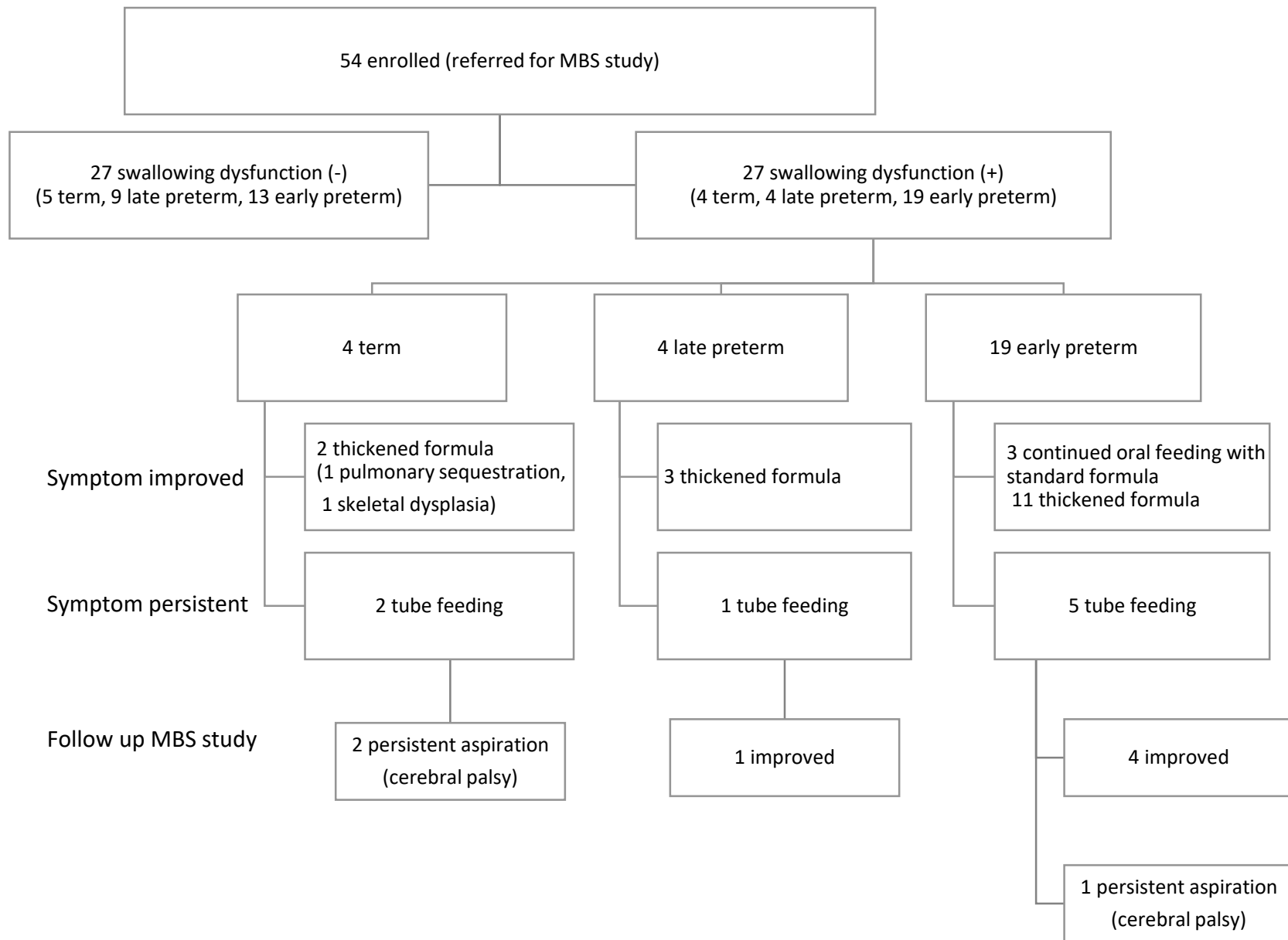

Supplement: Supplementary Materials — Patient flowchart. Among a total of 54 infants referred for MBS study, 27 infants had swallowing dysfunction. Four term infants with swallowing dysfunction had cerebral palsy. Two of them improved after dietary management with thickened formula. The remaining two infants continued tube feeding. Among four late preterm infants with swallowing dysfunction, three infants with normal development improved after dietary management with thickened formula. The remaining one infant with mild developmental delay continued tube feeding, who improved in follow-up MBS study. Among 19 early preterm infants with swallowing dysfunction, three infants who had only laryngeal penetration with mild feeding desaturation continued their oral feeding. Eleven infants improved after dietary management with thickened formula. The remaining five infants with severe swallowing dysfunction were discharged with tube feeding. Four of them improved in follow-up MBS study. One of them with cerebral palsy continued tube feeding. [file 5437376.f1.pdf]
